# Rapid but nondurable response of a *BRAF* exon 15 double-mutated spindle cell sarcoma to a combination of BRAF and MEK inhibitors

**DOI:** 10.18632/oncotarget.28606

**Published:** 2024-07-17

**Authors:** Kseniya Sinichenkova, Iliya Sidorov, Nataliya Kriventsova, Dmitriy Konovalov, Ruslan Abasov, Nataliya Usman, Alexander Karachunskiy, Galina Novichkova, Dmitriy Litvinov, Alexander Druy

**Affiliations:** ^1^Dmitriy Rogachev National Medical Research Center of Pediatric Hematology, Oncology, Immunology Ministry of Healthcare of Russian Federation, Moscow, Russia; ^2^Research Institute of Medical Cell Technologies, Yekaterinburg, Russia

**Keywords:** undifferentiated sarcoma, BRAF V600E mutation, low grade spindle cell sarcoma, abdominal cocoon

## Abstract

Introduction: BRAF V600E substitution predicts sensitivity of a cancer to BRAF inhibitor therapy. The mutation is rarely found in soft-tissue sarcomas. Here we describe a case of undifferentiated spindle cell sarcoma showing primary insensitivity to standard chemotherapy and pronounced but non-sustained response to BRAF/MEK inhibitors at recurrence.

Case presentation: A 13-year-old girl was diagnosed with low-grade spindle cell sarcoma of pelvic localization, BRAF exon 15 double-mutated: c.1799T>A p.V600E and c.1819T>A p.S607T in cis-position. The tumor showed resistance to CWS-based first-line chemotherapy and was treated surgically by radical resection. Seven months after surgery the patient developed metastatic relapse with abdominal carcinomatosis. Combined targeted therapy with BRAF/MEK inhibitors afforded complete response in 1 month and was continued, though complicated by severe side effects (fever, rash) necessitating 1–2 week toxicity breaks. After 4 months from commencement the disease recurred and anti-BRAF/MEK regimen consolidation was unsuccessful. Intensive salvation chemotherapy was ineffective. Empirical immunotherapy afforded a transient partial response giving way to fatal progression with massive, abdominal cocoon-complicated peritoneal carcinomatosis.

Conclusion: This is the first report of spindle cell sarcoma BRAF V600E/S607T double-mutated, responding to a combination of B-Raf and MEK inhibitors. Despite the low histological grade and radical surgical treatment of the tumor at primary manifestation, the disease had aggressive clinical course and the response to BRAF/MEK targeted therapy at recurrence was complete but nondurable. Empirical use of pembrolizumab provided no unambiguous evidence on the clinical relevance of immunotherapy in protein kinase -rearranged spindle cell tumors.

## INTRODUCTION

Undifferentiated sarcomas have unfavorable prognosis, as these tumors rarely harbor targetable aberrations and poorly respond to chemotherapy. The WHO Classification of Tumors, Soft Tissue and Bone Tumors, 5th Edition (2020) groups the undifferentiated sarcomas with aberrations of protein kinase-encoding genes into new entity termed NTRK-Rearranged Spindle Cell Neoplasms. The updated classification accounts for tumor biology rather than genome structure, as the clinically and pathologically distinct ETV6::NTRK3-rearranged infantile fibrosarcomas are regarded differentially. Instead, the group of NTRK-Rearranged Spindle Cell Neoplasms includes certain tumors driven by non-NTRK receptor and cytoplasmic kinases constitutively activated through genetic aberrations [1–3].

BRAF gene encodes a cytoplasmic serine-threonine kinase B-Raf constitutively activated in many cancers of various localization and histogenesis. BRAF-rearranged/mutated cases constitute 7% in cancer epidemiology. The most studied pathogenic variant BRAF V600E is found in all cases of hairy cell leukemia, up to 60% of skin melanomas and thyroid papillary carcinomas, and up to 10% of colorectal cancers. The prevalence of BRAF-mutated cases among soft-tissue neoplasms is tumor-specific, estimated as high as 15–20% for malignant peripheral nerve sheath tumors [4] and as low as 0.5–9% for undifferentiated sarcomas [5, 6].

In 2022, the US Food and Drug Administration (FDA) granted an accelerated approval to dabrafenib in combination with trametinib for unresectable or metastatic solid tumours with BRAF V600E mutation in patients older than 6 years having progression after prior treatment and no satisfactory alternative treatment options [7]. Several clinical cases of successful targeted therapy (TT) in patients with BRAF V600E-positive sarcomas have been published [8–16].

Here we describe a low-grade spindle cell sarcoma with double-mutated BRAF exon 15: c.1799T>A and c.1819T>A in cis-position, corresponding to V600E and S607T amino acid substitutions. Of note, the tumor tested as B-Raf V600E immunonegative (probably due to the second, neighboring S607T substitution) which interfered with the diagnostic algorithm and prioritized the role of molecular data in the ultimate diagnosis verification.

## CASE PRESENTATION

**Figure 1:**
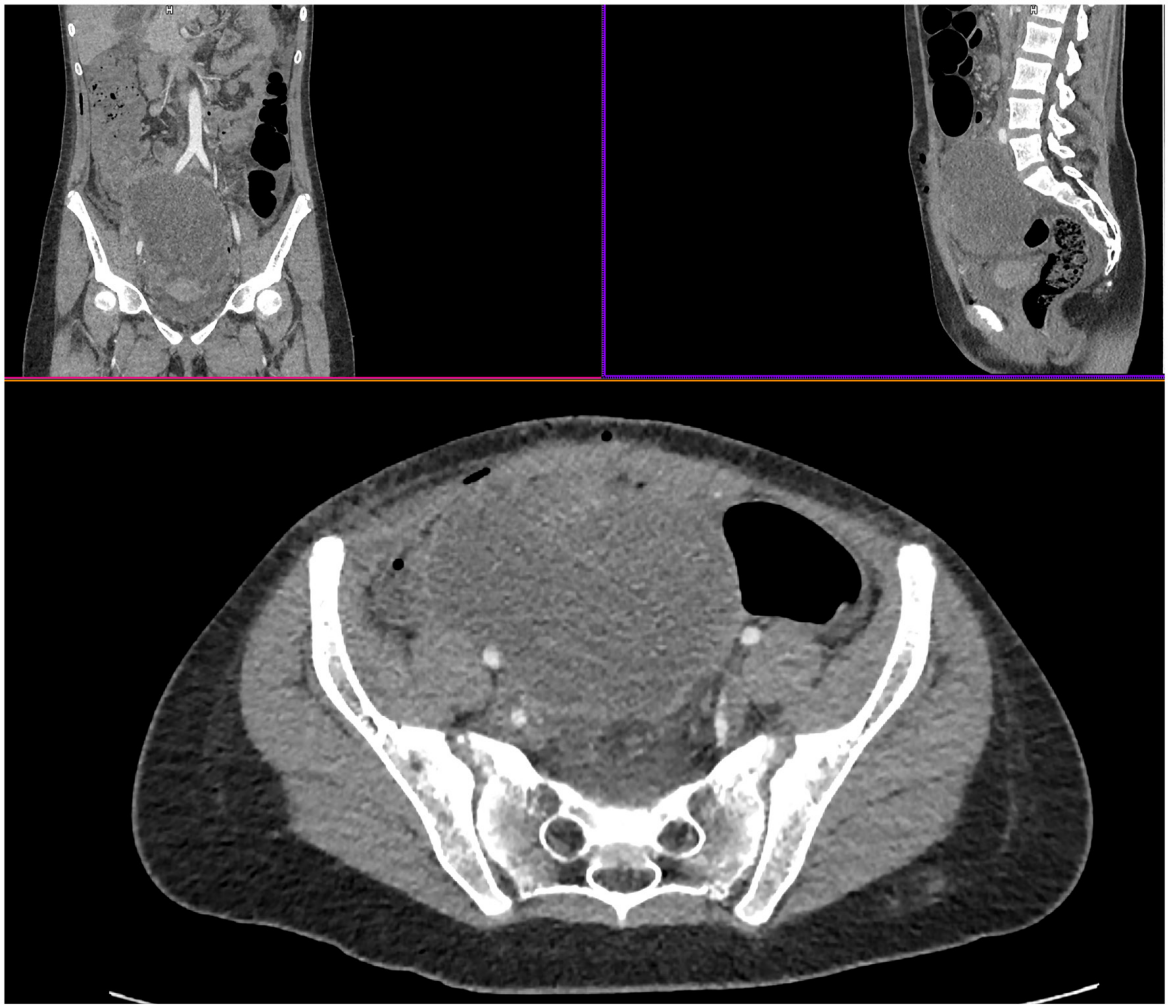
A multislice computed tomography of the original tumor. The image shows a distinctly outlined, moderately contrast-accumulating entity in the pelvic cavity at L5-S4 spinal level, without clear organ affiliation, causing displacement of adjacent structures.

A 13-year-old girl experienced paroxysmal abdominal pains increasing in dynamics and accompanied by hyperthermia. Laparotomy for suspected acute appendicitis revealed a large pelvic tumor protruding from the anterior abdominal wall and reaching 10 cm in diameter (Figure 1). A scanty biopsy of the tumor entailed a preliminary diagnosis of rhabdomyosarcoma. Examination revealed no signs of distant metastasis. After 2 courses of chemotherapy under CWS-2009 protocol (vincristine, doxorubicin, dactinomycin, ifosfamide) the neoplasm increased in volume from 581 cm^3^ to 757 cm^3^.

Due to the lack of response and adverse dynamics, it was decided to perform surgical resection of the neoplasm by low-transverse laparotomy. The intraoperatively visualized 13 × 12 × 10 cm tumor of bumpy appearance was adherent to the anterior abdominal wall, most likely originating from it, showing no clear signs of infiltrative growth at other surfaces. The tumor was removed by radical resection.

Morphological examination revealed a mass of small-to-medium spindle-shaped tumor cells forming compact patterns and short disordered fascicles in loose, locally edematous, fibrous stroma. Cell nuclei ovoid, regularly shaped, containing rough, unevenly dispersed chromatin and solitary irregular nucleoli; the cytoplasm unipolar, filamentous, homogeneous. The mitotic activity was low; specific features included adipose clusters within the tumor mass and abundant, relatively large blood vessels with hyalinosis zones at the periphery. The tumor was separated from surrounding tissues by thin pseudocapsule.

**Figure 2:**
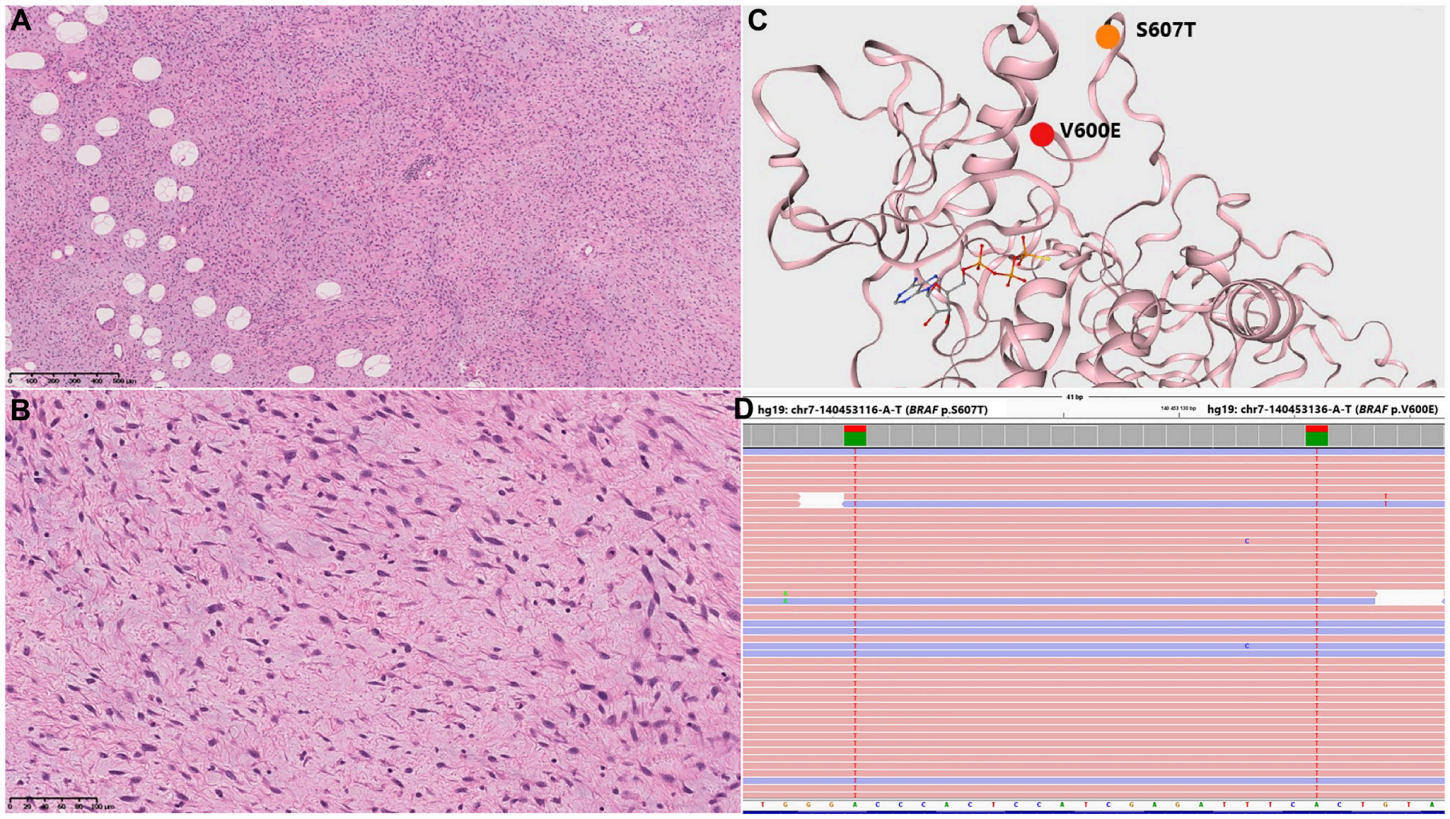
Morphological and genetic findings. (**A**, **B**) Representative histological images, H&E, ×50 (A) and ×200 (B), showing short fascicles of spindle-shaped tumor cells with large ovoid nuclei, fibrous stroma with diffuse focal myxomatosis, small adipose clusters and signs of perivascular lymphoid infiltration. (**C**, **D**) Two closely spaced missense mutations identified in BRAF exon 15: (C) Schematic representation of B-Raf protein structure (SwissModel) with amino acid substitutions at positions 600 and 607 of the polypeptide shown in red and orange, respectively; (D) an IGV screenshot demonstrating BRAF mutations c.1799T>A p.V600E and c.1819T>A; p.S607T in cis-position.

The sum of morphological features (spindle cell morphology, low cellularity and low-grade appearance, with adipose component and characteristic vascular patterns) entailed a suspected diagnosis of ‘spindle cell tumor with aberration in protein-kinase gene complex’. Such tumors are typically driven by NTRK rearrangements or BRAF V600E substitutions, immunohistochemically verified as positivity for corresponding protein markers. Meanwhile, immunohistochemical tests for TRK and B-Raf V600E, as well as EMA, MyoD1, Myogenin, CD117, DOG1 and STAT6, were negative (Figure 2A, 2B).

High-throughput sequencing of DNA isolated from the tumor and specifically enriched for putative oncogenic drivers and markers of solid tumors in children (the QIAseq customized panel protocol; Qiagen, Germany) revealed somatic double mutation in BRAF exon 15 (RefSeq NM_004333.6): c.1799T>A: p.V600E with 31% variant allele frequency (VAF) and c.1819T>A: p.S607T with 32% VAF in cis-position (Figure 2C, 2D).

**Figure 3:**
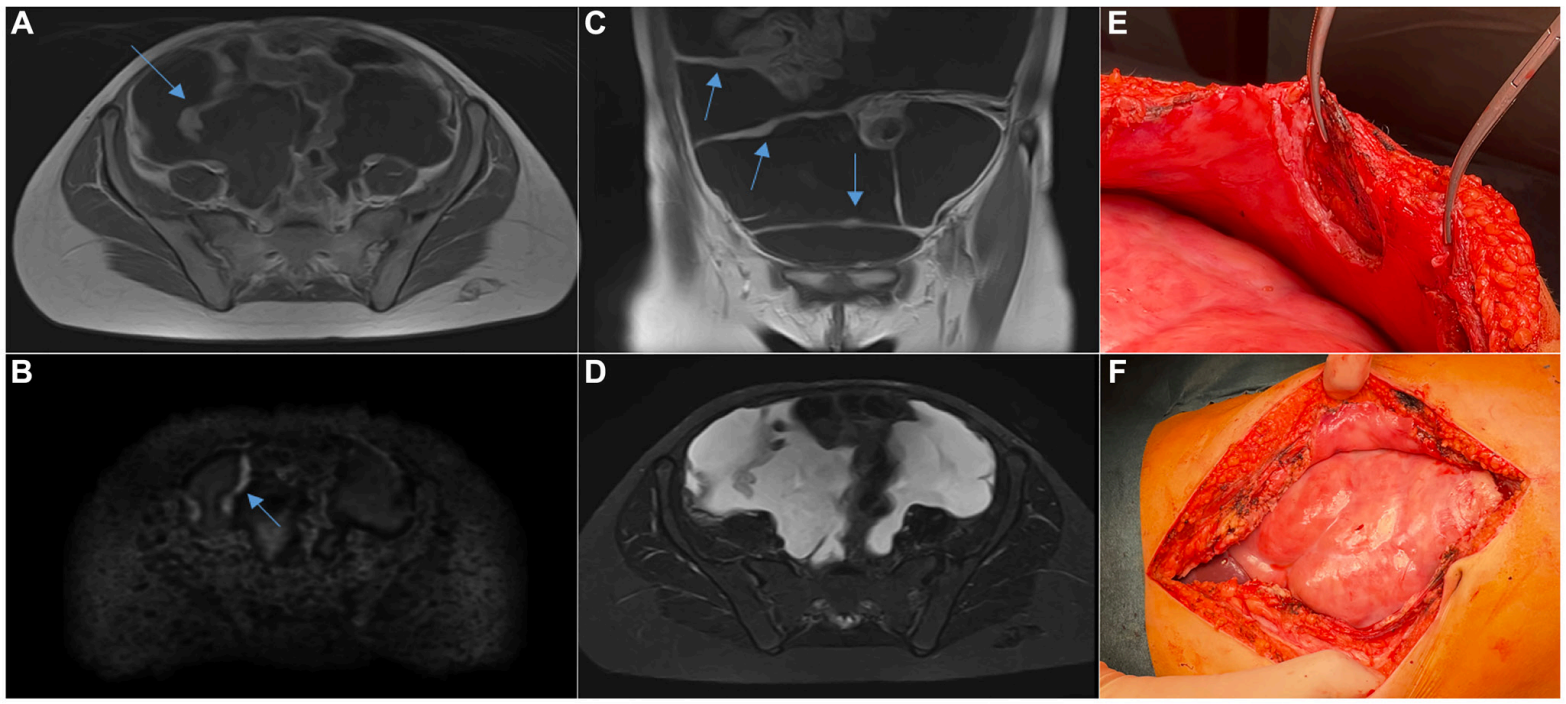
Peritoneal carcinomatosis during disease progression on immunotherapy. (**A**) T1 weighted image (WI), contrast-enhanced, axial plane; (**B**) DWI (b = 1000 c/mm^2^), axial plane; (**C**) T1WI contrast-enhanced, coronal plane; (**D**) T2WI STIR, axial plane; uneven thickening of the peritoneum against the background of ascites, visualized by contrasting, restricted diffusion; (**E**) intraoperative biopsy of the thickened peritoneum; (**F**) intraoperative view of the abdominal cocoon-encapsulated colon.

In view of the lack of response to first-line chemotherapy, the low histological grade of the tumor and the radical surgical treatment, the patient was discharged under observation. However, 7 months after surgery the patient started to experience abdominal pains and increase in abdominal circumference. MRI scans of abdominal and pelvic organs revealed peritoneal carcinomatosis with solid nodules and ascites indicating violent relapse of the disease (Figure 3A–3D).

A combination TT of vemurafenib 960 mg twice + trametinib 2 mg twice, daily, was commenced immediately. The choice of trametinib as MEK inhibitor was determined by unavailability of cobimetinib at the time of the treatment initiation. The therapy resolved the symptoms of ascites within 1 week and afforded complete regression of carcinomatosis and peritoneal foci as assessed by MRI 1 month from commencement. The dynamics amounted to complete therapeutic response; however, after 2 months the patient developed severe side effects (fever, diarrhea syndrome) necessitating regular toxicity breaks in the regimen for up to 7 days. Despite switching to a milder combination of dabrafenib 300 mg + trametinib 2 mg, daily, the toxicity persisted in the form of febrile fever with rash, necessitating toxicity breaks for up to 7 days and 25–50% reduction of doses for both components.

Eventually, the response was lost, as indicated by signs of ascites and carcinomatosis in control MRI 4 months from commencement. After the last toxicity break the regimen was consolidated to full doses, bringing alleviation of clinical symptoms (pain relief, reduction of abdominal circumference) in 2 weeks; however, in 2.5 weeks the patient developed fever, diarrhea, leukopenia and left-sided hydrothorax of about 1 liter requiring drainage of the pleural cavity. The inflammatory marker tests were negative; cytological examination and flow cytometry revealed no tumor cells in the evacuated pleural effusions; the hydrothorax was non-recurring and effectively resolved by the drainage. The complications were identified as treatment-related toxicity (of trametinib in particular); TT was discontinued and replaced with dexamethasone, 2 mg twice daily. Two days after TT discontinuation the toxicity symptoms subsided, which allowed resumption of TT with trametinib doses reduced to 50%, and no severe toxicity symptoms were further encountered.

**Figure 4:**
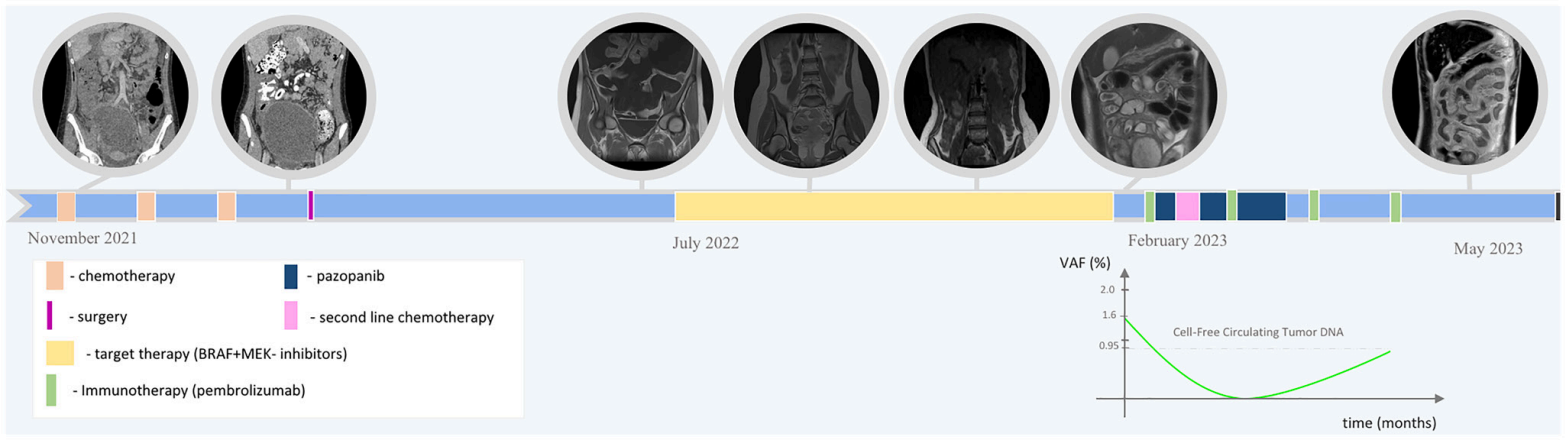
Overall treatment/monitoring timeline with a plot showing the circulating cell-free tumor DNA dynamics during immunotherapy.

The modified regimen afforded partial response; but after 1 month, MRI showed a soft-tissue nodule in the anterior abdominal wall, indicating carcinomatosis progression. Repeated surgical treatment/biopsy was contraindicated in connection with second-degree AB-blockade developed by the patient. The ascites fluid was cytologically clear of tumor cells, but contained cell-free tumor DNA BRAF V600E-mutated (VAF = 47%) as measured by digital droplet PCR tests, whereas cell-free DNA circulating with peripheral blood contained the mutant allele at VAF = 1.6% (Figure 4).

Salvage chemotherapy (carboplatin 200 mg/m^2^/day 4 days, etoposide 100 mg/m^2^/day 4 days) was ineffective, as the clinical signs of carcinomatosis continued to aggravate. In view of the lack of other options the regimen was switched to empirical immunotherapy with pembrolizumab 2 mg/kg + pazopanib 800 mg daily. Two courses of pembrolizumab afforded positive dynamics: 82% reduction of the nodule at 0.27% BRAF V600E allele frequency in circulating cell-free DNA. The 3rd course of pembrolizumab afforded clearance of the mutant allele from the cell-free circulating fraction; however, after the 4th course the abdominal carcinomatosis progressed against 14% reduction of the tumor nodule and cell-free mutant allele copies started to circulate with the blood (VAF = 0.95%). The patient underwent laparotomy revealing a massive adhesion process encompassing the entire visceral peritoneum with abdominal cocoon formation and parietal layer thickening up to 1 cm (Figure 3E, 3F). Histological examination revealed vital tumor tissue in the parietal peritoneum, histologically identical to the original tumor. The therapy was discontinued. One month later the patient died of disease progression.

## DISCUSSION

The prevalence of particular molecular driver events is known to depend on cancer type. BRAF V600E substitutions are typical for a variety of cancers including skin melanoma, but rarely found in sarcomas [17, 18]. Among soft-tissue cancers, this pathogenic variant is found in 12–20% of malignant peripheral nerve sheath tumors and estimated 3.5–13.5% of primary KIT/PDGFRA wild-type gastrointestinal stromal tumors [19, 20]. Activating genetic events in BRAF (missense substitutions, rearrangements) have been also described in infantile fibrosarcomas and tumors classified as NTRK-Rearranged Spindle Cell Neoplasms by the 5th Edition of the WHO Classification of Tumors (2020) [21, 22].

A low-differentiated soft-tissue tumor composed of spindle-shaped cells can be difficult to differentially diagnose from metastatic melanoma. These neoplasms are morphologically similar, with the likely fusiform cell shapes and possible lack of immunohistochemical determinants of melanocytic differentiation in melanoma metastases. Accordingly, BRAF-mutated soft-tissue sarcomas are often mistaken for metastases of dedifferentiated (nonpigmented) melanoma [23].

The current case of undifferentiated sarcoma in adolescent was diagnostically challenging as its BRAF V600E-positive mutation status was not accompanied by corresponding immunopositivity. Such BRAF double-mutated cases are rare and corresponding clinical details are missing. The rare S607T substitution was previously reported in a unique case of colorectal cancer [24] and its pathogenic role is uncertain, whereas BRAF V600E is a canonical driver event of tumorigenesis. The lack of immunoreactivity with the B-Raf V600E mutation-specific antibody can be explained by altered conformation of the protein conferred by the extra amino acid substitution, S607T, in the vicinity of position 600.

The current case of spindle cell soft-tissue sarcoma exemplifies a misleading incongruence between the low-grade histological appearance of the tumor and its recurrent violent progression with peritoneal dissemination. Many low-grade sarcomas are chemo- and radiation therapy-resistant, but spontaneous growth rates of such tumors are usually low. In the global clinical experience, the use of TRK inhibitors in children and young adults with NTRK-rearranged spindle cell tumors afford a prompt and durable response in up to 96% of the cases [25–27]. The rapid development of resistance to BRAF/MEK inhibitors in the current case may be associated with BRAF S607T as a primary resistance factor supporting the constitutive activity of B-Raf V600E. This suggestion is indirectly supported by ambiguous pathogenic significance of the S607T substitution revealed in silico.

On the other hand, BRAF-mutated melanomas typically develop resistance to targeted monotherapy with B-Raf inhibitors within 1 year from commencement, and some of them are primarily resistant to the drugs (vemurafenib, dabrafenib) [28, 29]. There are multiple routes of tumor resistance to B-Raf inhibition, involving about a dozen specific mechanisms, and several routes can develop simultaneously in one patient.

Current clinical standards for BRAF-mutated melanoma use a combination of B-Raf and MEK inhibitors. According to substantive high-quality evidence from randomized comparative studies, this combination prolongs both progression-free and overall survival rates compared with B-Raf inhibitor monotherapy [30–32]. Of note, patients with melanoma progression against B-Raf inhibitor monotherapy may benefit from a combination of B-Raf/MEK inhibitors [33], while anti-MEK monotherapy against the background of anti-B-Raf TT resistance is non-efficacious. Despite the two-point inhibition of the RAS-RAF-MEK signaling cascade at the level of mutation-activated B-Raf and the downstream MEK proteins, the majority of patients develop resistance to vemurafenib + cobimetinib and dabrafenib + trametinib combinations within 3 years. The mechanisms of resistance to combined anti-B-Raf/MEK regimens are similar to those described for anti-B-Raf monotherapy and involve mutations in MEK genes (MAP2K1, MAP2K2, etc.) and BRAF amplifications [34]. In the current clinical case, the patient repeatedly responded to anti-B-Raf/MEK TT, albeit the second response was partial and extremely transient.

The paradigm of non-efficacy of immunotherapy in soft-tissue sarcomas was challenged over the recent 5 years, as certain histological types of sarcoma were shown to respond to the immune checkpoint inhibition. Alveolar sarcomas were shown to respond to atezolizumab in one-third of the cases; the protocol was approved for clinical use in 2022. In SARC028 multicenter phase II study, pembrolizumab showed clinical efficacy against undifferentiated pleomorphic sarcoma and pleomorphic liposarcoma in 23% and 10% of the cases, respectively [35].

The clinical presentation of abdominal cocoon aka idiopathic or primary sclerosing encapsulating peritonitis is rather untypical for carcinomatosis. Abdominal cocoon is a multifactorial clinical phenomenon classified into primary (idiopathic, causative event unknown) and secondary (caused by peritoneal dialysis, chronic abdominal/pelvic inflammation, tuberculosis, sarcoidosis or autoimmune conditions, as well as B-blocker or chemotherapy complications) [36, 37]. The uniform thickening of parietal and visceral peritoneal layers accompanied by massive adhesion process was observed intraoperatively. Specific carcinomatous nature of these changes was confirmed histologically.

## CONCLUSIONS

This is the first report of spindle cell sarcoma BRAF V600E/S607T double-mutated, responding to a combination of B-Raf and MEK inhibitors. Despite the low histological grade and radical surgical treatment of the tumor at primary manifestation, the disease had aggressive clinical course and the response to BRAF/MEK targeted therapy administered at recurrence was complete but nondurable.

The case demonstrates the urgency of comprehensive tumor DNA/RNA testing for verification of the diagnosis and selection of targets for molecular-oriented therapy. TT administered in accordance with identified predictive marker had prompt and profound effects amounting to complete therapeutic response. However, the emerging resistance, apparently backed by toxicity breaks in the regimen, led to eventual loss of therapeutic response and violent progression. Consolidation of TT regimen provided an extremely transient partial response of the recurrent tumor. Empirical use of pembrolizumab provided no unambiguous evidence on the clinical relevance of immunotherapy in protein kinase - rearranged spindle cell tumors.

## Abbreviations

FDA: Food and Drug Administration
VAF: variant allele frequency
